# Perceived stress, resilience, well-being, and COVID 19 response in Isha yoga practitioners compared to matched controls: A research protocol

**DOI:** 10.1016/j.conctc.2021.100788

**Published:** 2021-05-21

**Authors:** P. Upadhyay, S. Narayanan, T. Khera, L. Kelly, P.A. Mathur, A. Shanker, L. Novack, S. Sadhasivam, K.A. Hoffman, R. Pérez-Robles, B. Subramaniam

**Affiliations:** Oregon Health & Science University, United States

**Keywords:** Yoga, Meditation, COVID-19, Perceived stress, Isha

## Abstract

**Objectives:**

The COVID-19 pandemic has been a significant stressor worldwide and reports of psychological distress, depression, sedentary lifestyles, and overall decreased wellbeing are increasing. Yoga practices have been found to improve mental and physical health. The purpose of this randomized controlled trial is to compare Isha yoga practitioners to controls on perceived stress, resilience, wellbeing, and protection and recovery from COVID-19. Trial Design. In this prospective randomized control trial, the effects of yoga practices are being compared between seasoned yoga practitioners with two controls who are age (±3 years), gender matched, and living in the same neighborhood.

**Methods:**

Participants will be asked to complete a series of web-based surveys at baseline, six weeks, and 12 weeks. These surveys include validated scales and objective questions on COVID-19 infection and medical history. The validated questionnaires assess stress, mood states, resilience, and overall wellbeing. Questionnaires, weekly activity diaries, and medical history, will be collected using REDCap.

**Results:**

We hypothesize that routine yoga practice during the COVID-19 pandemic will reduce stress, enhance well-being, and provide protective effects against COVID-19.

**Conclusion:**

With the growing concern about the physical and mental impacts of COVID-19 and increased interest in alternative practices such as yogic practices, this study will contribute to the growing body of evidence about the safety and efficacy of yoga for emotional, mental, and physical health conditions.

## 1 Introduction

With nearly 82 million cases and 1.8 million deaths worldwide as of the end of December 2020 [1], the COVID-19 pandemic has been a significant stressor worldwide. The severity of the disease and dearth of definitive treatments has led to thousands of deaths each day. Our understanding of the pandemic's effects on individuals, families, and communities as well as our methods of coping continue to evolve. Measures such as social distancing and working remotely have been enforced worldwide to curb disease transmission. This has led to an increased number of people staying indoors, living a more sedentary lifestyle, and greater feelings of isolation [2]. Additionally, reports of psychological distress [3,4], anxiety [5], sleep disturbances [6], and problematic substance use have increased [7]. In the U.S., the COVID-19 pandemic is exacerbating the opioid epidemic and has led to a dramatic increase in overdose deaths [8]. Clinicians and public health professionals have stressed the importance of maintaining good mental, emotional, and physical health during these turbulent times [9,10].

Yoga practices, which include specific breathing, meditation, and posture protocols, can enhance physical and mental well-being [11,12]. Research demonstrates that regular yoga practice can modulate the autonomic nervous system [13], improve sleep quality [14], boost immunity [15] and reduce stress [16], anxiety [17,18], and depression [19]. Regular yoga practice also has demonstrated neuroprotective effects [20] as well as benefits for cardiac autonomic control and breathing function [21]. In addition to these well-being indicators, there is some evidence that yoga practices can improve immune, lung, and respiratory function and enhance anti-inflammatory response – important factors for COVID-19 protection and recovery. In two systematic reviews of yoga and asthma, researchers found some evidence that yogic practices can reduce medication usage and improve symptoms [22,23]. In a study of respiratory illnesses, the meditation group compared to two control groups (exercise or no intervention), was found to suffer less severe symptoms [24]. Similarly, Barrett et al. [25], sought to investigate potential preventive effects of meditation or exercise on incidence, duration, and severity of acute respiratory infection (ARI) illness. They found that compared to the control or exercise groups, illness duration, number of days-of-work missed, and illness severity was less for the meditation group.

Yoga can be practiced by anyone, requires no infrastructure, and is a restorative, individual activity easily performed during periods of social distancing. Public interest in yoga practices is increasing and often touted in media outlets as a means of coping with stress and improving health [[26], [27], [28]]. The Centers for Disease Control and Prevention reports that between 2012 and 2017, the number of adults practicing yoga or meditation rose from 9.5% to 14.3%, and 4.1% to 14.2 respectively [29]. More rigorous research is needed to better understand how yoga may play a role in improving mental and physical well-being in the general population.

## **2** **Objectives.** The purpose of this randomized controlled trial is to accomplish the following specific aims

Specific Aim 1: To quantitatively assess the effects of Isha yoga practices between seasoned practitioners and control groups on perceived stress, resilience, and wellbeing by use of validated scales.

Specific Aim 2: To compare protection and recovery from COVID-19 infection between seasoned Isha yoga practitioners and controls as demonstrated by:

a. Self-reported duration of fever and respiratory symptoms in COVID-19 positive participants
b. Self-reported readiness to return to work (or a feeling of being physically and mentally fit)

Specific Aim 3: To compare prevalence rates of diagnosed COVID-19 infection between the seasoned Isha yoga practitioners and age and gender matched controls at baseline, 6 weeks and 12 weeks.

We hypothesize that routine yoga practice during the COVID-19 pandemic will reduce stress and enhance well-being.

## 3 Materials and methods

### 3.1 Participant recruitment

We will employ a two-stage recruitment design. First, seasoned yoga participants will be recruited by social media, websites, flyers, word of mouth, email announcements at the Isha Foundation [30], an international school of yoga. Seasoned yoga participants are defined as individuals who have completed at least one, in-person yoga Isha Yoga training protocol with an Isha yoga trainer and are regularly doing the practice. The Isha Foundation will facilitate this stage of the recruitment by including study recruitment flyers and links to its broad follower base of meditators and yoga practitioners. These communications will have a survey link where prospective participants can confirm their interest in the study and learn more detailed information about study procedures. They will then be directed to a brief REDCap survey where they will be asked screening questions to ensure study eligibility. Those who are eligible will be directed to an electronic consent form and enrolled in the study following the submission of the consent agreement.

The second stage of the recruitment uses snow-ball sampling [31]. Following enrollment in the study, seasoned yoga practitioners are requested to nominate two friends who can act as age and gender matched controls, preferably living in the same neighborhood. The study team will reach out to the nominated individuals to describe the study, assess interest and eligibility, and direct prospective respondents to an electronic consent form specific for the control groups. Respondents will be enrolled following the provision of their consent.

The process of nominating controls for the study is voluntary and seasoned yoga practitioners can participate without referring any controls. In this situation, unmatched controls and practitioners will be matched by the study team based on their self-reported location details. The team prioritizes matching the zip codes followed by town, city, county, and state.

### 3.2 Intervention description and timing

This is analytic study wherein an observational arm of ‘seasoned yoga practitioners’ is being compared with age, gender, and region matched ‘non-meditators’ as controls. The non-meditators control group is further randomized to active comparator arm and placebo comparator arm.

Control participants or Non-meditators are individuals with minimum (<1 month) to no yoga practice of any kind, prior to study enrollment. Non-meditator controls randomized to an ‘Active comparator arm’ are taught a 3 min yoga practice (Simha Kriya) which involves deep breathing exercises and meditation following randomization. Within the yogic tradition, there are breathing exercises which are considered to foster pranayama, which builds a type of “armor” or Kavacham for oneself against negative impacts encountered in the every day world. Prana is considered the fundamental energy and the foundation for all life. Pranayanic practices have been shown to contribute to positive physiologic and psychologic changes including improvements in hypertension, cognitive function, breathing frequency, stress, and heart rate variability [13,[32], [33], [34]]. In a study of another Isha pranayama practice, Peterson et al. found that after 6 weeks of daily practice, participants reported improved general well-being (General Well-Being Scale) and lower levels of perceived stress (Perceived Stress Scale) [35]. In the current study, the Simha Kriya practice will be taught on-line by highly-trained Isha practitioners and participants are asked to perform Simha Kriya using a web-based application, twice per day.

**Fig. 1:**
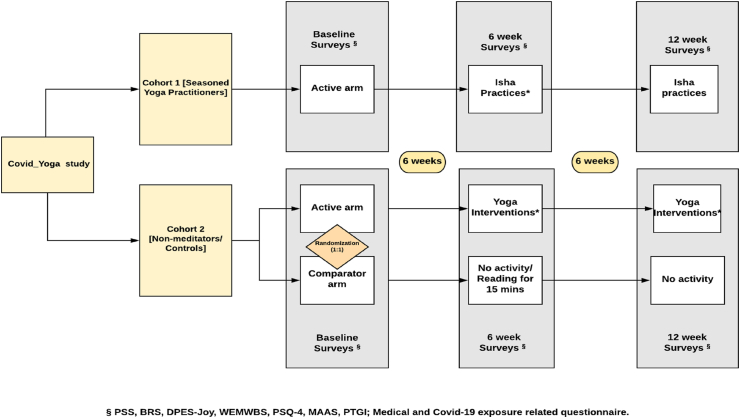
Study flow chart.

The other control group acts as a ‘Placebo comparator arm’ and performs either reading activities or remaining idle for 15 min a day throughout the study period. Respondents in the observational arm (seasoned yoga practitioners) continue their usual yoga practices. Please see Fig. 1 for study flow chart. Participants aged <18 years or >80 years will excluded from the study. Please see Table 1 for additional Inclusion/Exclusion Criteria.

**Table 1:** Inclusion and exclusion criteria.

	Inclusion	Exclusion
Seasoned Yoga Practitioners	Must be an Isha School of Yoga practitioner	
	Must have completed Inner Engineering Online course and practice Shabhavi Mahamudra Kriya or Shakthi Chalan Kriya	
		Participants <18 years of age
		Participant >80 years of age
		Participants must be able to read and understand English.
Control groups	Must have little to no experience with Isha school of yoga	
	Should have practiced yoga routinely for <1 Month (if previously experienced in yoga)	
		Participants < 18 years of age
		Participant > 80 years of age
		Participants must be able to read and understand English.

### 3.3 Data collection

Participants will be asked to complete a series of web-based surveys at baseline, six weeks and 12 weeks. These surveys include validated scales and objective questions on COVID-19 infection and medical history. The validated questionnaires assess stress, mood states, resilience, and overall wellbeing. Questionnaires, weekly activity diaries, and medical history, will be collected using REDCap. All surveys will all be automated and the study team will have minimal interaction with the participants. All health information provided by the participants is self-reported.

### 3.4 Measures

Each participant will be asked to respond to 8 validated neuropsychological scales assessing stress (Perceived Stress Scale, PSS), anxiety and depression (PHQ-4), joy predisposition (DPES-Joy Subscale), mindfulness awareness (MAAS), resilience (BRS), mental well-being (WEMWBS), and post-traumatic growth (PTGI). PSS is defined as the primary outcome while BRS is the key secondary outcome.

**Questionnaires**. The following questionnaires are incorporated into the study survey and will be administered to the study participants:

*Perceived Stress Scale (PSS).* PSS is a 10-question validated instrument that assesses stress. Participants are asked to rate on a scale of 0 (never) to 4 (very often) how often they agree with various statements designed to assess the degree to which life situations are appraised as stressful. Items from each of the 10 questions are then summed to create a total perceived stress score.

*4 Item-Patient Health Questionnaire measuring Anxiety and Depression (PHQ-4).* Four questions rated on a scale of 0 (Not at all) to 3 (Nearly every day). The sum is computed for the responses and scores range from 0 to 12. Categories of psychological distress are classified as: None (0–2 score), Mild (3–5 score), Moderate (6–8 score) and severe (9–12 score). Anxiety subscale is calculated by measuring sum of item 1 and 2 (score range, 0–6; scores greater than 3 considered positive for anxiety screening.) Depression subscale is calculated by measuring sum of item 3 and 4 (score range, 0–6; scores greater than 3 considered positive for depression screening.)

*Dispositional Positive Emotion Scale- Joy Subscale (DPES-Joy).* The joy subscale of DPES is a 6-item questionnaire that measures a dispositional tendency to feel joy in life. Participants report their level of agreement to each item on a scale of 1 (Strongly agree) to 7 (Strongly disagree) and a mean score is calculated.

*Mindfulness Attention Awareness Scale (MAAS)- short form.* MAAS - short form is a 5-question validated instrument that assesses mindfulness and attention while performing routine activities of daily living. Participants are asked to rate on a scale of 1 (Almost Always) to 6 (Almost Never) the frequency of occurrence for each experience. A mean score is then computed using items from each of the 5 questions to calculate the individual's MAAS score.

*Brief Resilience scale (BRS).* The BRS identifies an individual's ability to “bounce back” or recover from a stress. This 6-item scale is rated on a 5-point response scale (1 = Strongly disagree; 5 = Strongly agree). Responses from each of the 6 questions are summed to create a total score, producing a range of 6–30. The score is divided by the number of questions answered to calculate the individual brief resilience score.

*Warwick- Edinburgh Wellbeing Scale (WEMWBS).* The purpose of the WEMBS scale is to assess various aspects of mental health such as positive affect, satisfying interpersonal relationships, and positive functioning. This 14-item scale is rated on a 4-point response scale (1 = none of the time to 4 = all of the time). Response from each of the 6 questions is then summed to create a total wellbeing score.

*Post Traumatic Growth Inventory (PTGI).* The purpose of the PTGI is to capture the positive outcomes reported by a person who has experienced a traumatic event. This scale will be administered only to those participants who report being hospitalized during the COVID-19 pandemic. The scale helps to determine a respondent's coping ability in the aftermath of a trauma, their perceptions of self and others, and meaning of the event. This 21-item scale is rated on a 6-point Likert response scale and response to each question is summed to create a total PTGI score.

*Activity Diary.* The weekly activity diary is a tool which helps the participants to keep a track of their activities each week. This enables the study team to measure compliance and protocol adherence by the participants. Based on the construct of the study, we have 2 different activity diaries employed for data collection in this study. The activity diary for seasoned yoga practitioners collects information on their routine activity practiced, its frequency and duration. The activity diary for controls collects information on the activity suggested by study team (the Simha Kriya yoga practice vs placebo activity choices of reading or remaining idle).

### 3.5 Randomization

Non-meditators or control arm participants will be randomized into one of the two groups using the REDCap Block Randomization Technique. Participants will be randomized into one of two groups:

1) Active Comparator: Participants will be taught the 3 min Simha Kriya practice which involves deep breathing exercises with meditation (twice daily) for the study duration (12 weeks).
2) Placebo Comparator: Participants can choose to either read a book of their choice or stay idle for 15 min each day for a total duration of 12 weeks.

### 3.6 Analysis

Baseline characteristics of participants will be recorded. Continuous data will be presented as means ± standard deviation or median (interquartile range) depending on the distribution of the data and assessed with a parametric paired *t*-test or non-parametric Wilcoxon signed rank test, as appropriate. Normality will be assessed with the Shapiro-Wilk test. Categorical data will be presented as frequencies and percentages and assessed with a chi-square or Fisher's exact test, as appropriate. All primary analyses will be assessed using intention-to-treat principles. SAS 9.4 (SAS Institute Inc., Cary, NC) will be used for all analyses with two-sided p-values < 0.05 considered statistically significant.

Aim 1 Analysis. To quantitatively assess the effects of Isha yoga practices between seasoned practitioners and the control groups on measures such as perceived stress, resilience, and overall wellbeing by use of validated scales at baseline, 6 weeks and 12 weeks. We have defined Perceived Stress Score as our primary outcome and Brief Resilience Scale scores as our key secondary outcome. Given the continuous nature of the scales used to assess stress, resilience and measures of well-being, differences between groups will be assessed with a parametric or non-parametric *t*-test. Multivariable regression may be utilized to adjust for potential confounders that deem to vary between the groups based on the univariable analysis prior to that. The distribution of the dependent variable will depend on the actual distribution of the scales recorded in the study. A mixed effect approach in modeling will address the similarity of the subjects enrolled in the study as friends or by matching, by assigning a random intercept to each cluster of related subjects. To assess whether there are differences between baseline and at six weeks, we will employ the use of paired t-tests or non-parametric tests for univariable analysis, as appropriate. Additionally, the change in scales over time can be compared between the groups using mixed models approach, wherein an indicator of time (baseline/6 weeks) will define the direction and significance of the change in a model, adjusted independent of other factors.

Aim 2 Analysis. To Compare Protection and Recovery from COVID 19 infection between seasoned Isha yoga practitioners and controls as demonstrated by:

a. Self-reported duration of fever and respiratory symptoms in COVID-19 positive participants
b. Self-reported readiness to return to work (or a feeling of being physically and mentally fit)

We will calculate the protection and recovery rates from COVID-19 infection as defined by the aforementioned criteria. Given the continuous nature of the criteria used to assess symptom duration and sense of recovery, differences between groups will be assessed in a univariable manner with a parametric or non-parametric *t*-test. We will perform spot analysis at the three defined points to capture the point prevalence of recovery rates. We also intend to assess the differences between the prevention and recovery rates at baseline and six weeks and baseline and twelve weeks. We will employ the mixed model technique similarly to the analysis suggested in the aim #1 analysis.

Analysis of Aim 3: To compare Prevalence rates of diagnosed COVID-19 infection between the seasoned Isha yoga practitioners and age and gender matched controls at baseline, 6 weeks, and 12 weeks.

We will compute period prevalence of diagnosed COVID-19 infections as a measure of frequency. Since the data to be analyzed are not dependent on duration, we intend to compute the prevalence rates at the following time points: baseline, 6 weeks, and 12 weeks. The result would be reported as the portion of study population with COVID-19 at the specified time point.

Finally, we will perform an exploratory analysis of the data collected by comparing the effect of yoga practices and their duration between the 3 study groups to estimate a dose-response trend if possible.

Compliance is defined as 3 days of activity each week for at least 3 weeks from baseline to 6 weeks, or a minimum of 6 weeks from baseline to 12 weeks. Measures of frequency and association will also be calculated. We will complete exploratory analysis by comparing the different yoga practices and their duration between the 3 study groups to assess a dose-response pattern.

After reviewing the data that was collected over the 12 week period, several amendments were made to the protocol. For example, we compared the three groups, Group 1: Simha-Kriya Yoga for a period of 12 weeks, Group 2: Reading activity/Sitting idle for a period of 12 weeks, and the Yoga Practitioners, rather than the combination of control groups with the yoga practitioners. We also performed Poisson regression rather than linear regression to account for potential confounders due to the distribution of the primary outcomes, PSS and BRS scores. These changes were towards a more conservative approach which we believe provided more valid inference.

## 4 Discussion

The COVID-19 pandemic has placed significant stress on individuals, families, and communities. Psychological distress, depression, sedentary lifestyles, and decreased overall wellbeing are on the rise. Even prior to the pandemic, depression and anxiety were the leading source of adult disability worldwide [36,37]. Yogic practices which include breathing, meditation, and postures, have shown promise as a mechanism for improving mental and physical health. In a meta-analysis of 41 trials, researchers found evidence that meditation reduced multiple negative dimensions of psychological stress such as anxiety and depression [38]. In a systematic review [39] which investigated the effects of yoga on sympathetic nervous system and hypothalamic pituitary adrenal axis regulation measures, evidence suggested that yoga practice led to improved regulation of these systems and decreased depression and anxiety symptoms. Similarly, in a recent study of a yogic breathing protocol on emotion processing, anxiety, and affect, thirty individuals were assessed at baseline and after 4 weeks of practice [40]. Functional magnetic resonance imaging (MRI) showed significantly decreased states of anxiety and negative affect at follow-up, and the modulation of activity in brain regions involved in emotional processing, attention, and awareness.

In this study, we will compare the effects of yoga practices between seasoned yoga practitioners and two controls who are age ( ±3 years) and gender matched and living in the same neighborhood. This randomized controlled trial will compare seasoned yoga practitioners to non-meditators who have been randomized into active comparator vs placebo comparator arms on various outcome measures including perceived stress, resilience, wellbeing, and protection and recovery from COVID-19. Though time and funds were limited for this particular study, in the future we hope to include additional wellness measures such as sleep duration and quality, as well as objective measures of compliance such as Fitbit.

We hypothesize that routine yoga practice during the COVID-19 pandemic will reduce stress, enhance well-being, and provide protective effects against COVID-19. While a direct comparison between seasoned yoga practitioners and non-meditators at baseline would have been sufficient to establish the effectiveness of yoga practices; the dynamic progression of COVID in varying geographical regions at different rates warranted these comparisons at least at two other time points separated by 6 weeks each. Furthermore, by incorporating a robust short (3 min) breathing yoga activity into the non-meditator cohort in a randomized fashion, we aimed to establish that exposure to any yoga practice will significantly improve mental health status in non-meditators. With the growing concern about the physical and mental impacts of COVID-19 and increased interest in alternative practices such as yogic techniques, this study will contribute to the growing body of evidence about the safety and efficacy of yoga for emotional, mental, and physical health conditions.

## Author contribution

SB, PU, LN: Conceptualization; NS, KT, PM, AS: Data curation; PU, LN, LK: Analysis; SB, PU, LN, SS*:* Methodology; PU: Project administration/Supervision; All authors*:* Writing; All authors: Review and editing.

## Trial registration

ClinicalTrials.gov trial identification number NCT04498442.

## Declaration of competing interests

The authors declare that they have no known competing financial interests or personal relationships that could have appeared to influence the work reported in this paper.
